# An ALoGI PU Algorithm for Simulating Kelvin Wake on Sea Surface Based on Airborne Ku SAR

**DOI:** 10.3390/s25144508

**Published:** 2025-07-21

**Authors:** Limin Zhai, Yifan Gong, Xiangkun Zhang

**Affiliations:** 1 Key Lab of Microwave Remote Sensing, National Space Science Center, Chinese Academy of Sciences, Beijing 100190, China; zhailimin21@mails.ucas.ac.cn (L.Z.); gongyifan22@mails.ucas.ac.cn (Y.G.); 2 School of Electronic, Electrical and Communication Engineering, University of Chinese Academy of Sciences, Beijing 100048, China

**Keywords:** airborne SAR, Ku band, Gaussian and Laplacian, iteration, PU, Kelvin wake, sea-ship surface height, wave height inversion

## Abstract

The airborne Synthetic Aperture Radar (SAR) has the advantages of high-precision real-time observation of wave height variations and portability in the high frequency band, such as the Ku band. In view of the Four Fast Fourier Transform (4-FFT) algorithm, combined with a Gaussian operator, a Laplacian of Gaussian (LoG) Phase Unwrapping (PU) expression was derived. Then, an Adaptive LoG (ALoG) algorithm was proposed based on adaptive variance, further optimizing the algorithm through iteration. Building the models of Kelvin wake on the sea surface and height to phase, the interferometric phase of wave height can be simulated. These PU algorithms were qualitatively and quantitatively evaluated. The Principal Component Analysis (PCA) scores of the ALoG iteration (ALoGI) algorithm are the best under the tested noise levels of the simulation. Through a simulation experiment, it has been proven that the superiority of the ALoGI algorithm in high spatial resolution inversion for the sea-ship surface height of the Kelvin wake, with good stability and noise resistance.

## 1. Introduction

The airborne SAR is an aerial radar system that can not only observe the water surface in space and time, but also has convenient installation and low maintenance costs compared to a space-borne radar. Tings et al. [1] and Graziano et al. [2] have confirmed that sensors with shorter slant ranges and X-band are more suitable for detecting ship wakes by comparing space-borne Sentinel-1 (C-band), RADARSAT-2 (C-band), TerraSAR-X (X-band), and CosmoSkymed (X-band) SAR data. Short-wave high-frequency electromagnetic waves (K-band) have strong penetrating capabilities, making them more conducive to measuring the Kelvin wake wave height. Hwang et al. [3] and Carrasco et al. [4] were able to obtain significant wave height from the variations in Doppler speed measured by X-band radar. A physics-based methodology was further developed based on this, which can be used in any sea state conditions without calibration [5]. Liu et al. [6] conducted a simulation of SAR imaging of dynamic wakes of a submerged body, which can significantly simplify scattering computation and image formation. In the complex images of radar, amplitude represents the echo intensity of the target, which can be used for target detection.

The research on ship wakes can be traced back to the 19th century [7], which has important reference values for ship detection and classification [8], and also provides ideas and methods for submerged body detection. Lord Kelvin is a pioneer in ship theory, first proposing the principle of the formation of surface waves on a moving target ship on the sea surface, and based on this, he discussed the wake problem of point disturbances in hydrostatic water [9]. The Kelvin wake (divergent and transverse waves) is a V-shaped wake formed by surface gravity waves with an angle of approximately 39° around the ship. The Kelvin wake of the ship in radar images is often more pronounced than that of a ship with a low Radar Cross Section (RCS). Shemer et al. [10] proposed a mathematical model to simulate wave images in ship wakes using either regular or airborne interferometric SAR (InSAR). Zilman et al. [11] investigated the detection of Kelvin wakes in simulated SAR images and assessed the influence of significant wave height on their detectability. Jia et al. [12] simulated the received radar echo signals and used SAR imaging algorithms to focus the SAR raw signals of the wakes, generating SAR images of the wakes. The ship wake in SAR images is determined by the observation system parameters, ship parameters, and environmental sea conditions. These factors cause difficulties for the analysis and interpretation of true radar images. Hennings et al. [13] compared SAR images from different satellites with simulated Kelvin arms, finding that the Kelvin arm’s radar signal is strongest at low wind speeds and not very sensitive to wind direction. Zhao et al. [14] developed a Bistatic SAR (Bis-SAR) image intensity model for a composite ship–ocean scene, demonstrating the contribution of the interaction effect between ships and the sea surface to the Bis-SAR intensity. Bao et al. [15] proposed airborne Along Track InSAR (AT-InSAR), demonstrating that AT-InSAR phase images are more suitable for measuring ocean wave spectra than traditional SAR images. In this paper, the Michell thin-ship theory [16] based on the model of Kelvin wake and the JONSWAP [17] wave spectrum are used to simulate airborne radar images corresponding to ambient sea waves and superimposed ship wakes.

The interferometry of the phases measured twice can be used to invert micro deformations and extract deformation features of the monitored areas with sub-millimeter precision [18]. Eshbaugh et al. [19] confirmed the fine sensitivity of the interferometric technique to measure surface wave heights. The PU step is essential in radar interferometry. Zeng et al. [20] unwrapped the time series of the electromagnetic echoes’ phases from a one-dimensional (1D) wavy ocean surface simulated, confirming the effectiveness of estimating wave height using phase time series. Sun et al. [21] solved multiple collectively accumulative wrapped phase problems based on the Transport of Intensity Equation (TIE) from a two-dimensional (2D) perspective, and used an iterative strategy to enhance the performance of the TIE-based method. Zhao et al. [22] estimated the analytical solution based on the TIE, which is widely used for solving the equation. However, PU can only be successfully achieved in very low noise conditions, and further iterations are needed in practical applications to mitigate the impact of noise. Wang et al. [23] combined deep learning to improve TIE. The deep learning TIE (DTIE) highlights that only one input intensity image is needed, which is not affected by image boundary problems and is insensitive to noise. However, due to the loss function of the neural network not converging to zero, some detailed information is lost. The variance of phase error mainly depends on noise variance, intensity modulation, and fringe density. In addition, the combination of similar noise sources usually manifests as Gaussian noise sources [24]. So, many distributions can be approximated by Gaussian distributions. These existing studies have provided ideas for the inversion of a ship’s Kelvin wake wave height on the sea surface; therefore, research has been conducted on the parameters of airborne radar systems, as well as the PU algorithms for 2D interferometric phase.

The rest of this article is organized as follows: Section 2 introduces the Kelvin wake wave height of the ship, Kelvin wake on the sea surface, and the model of the sea-ship surface height to phase. The ALoG is derived with the principle of iteration, and the experiment on simulated interferometric phase images is conducted to validate the proposed methods, which are presented in Section 3. The discussion of PCA, profile analysis, and sea surface background in Section 4. Conclusions are drawn in Section 5.

## 2. Simulation of Kelvin Wake on the Sea Surface

### 2.1. Kelvin Wake Wave Height

In radar images, the turbulent wake of a ship is easier to identify compared to the ship itself. Among them, the Kelvin wake is the most common type of wake structure. The Kelvin wave system includes transverse and divergent waves, as well as cusp waves formed by wake edge interference, which appear as sharp lines on radar images, known as Kelvin envelopes and Kelvin arms. So, the Kelvin wake modeling method was used to simulate wave height.

Assume that water is non-viscous and non-compressible and that the hull takes on a Wigley parabolic shape [25]. Establish a coordinate system, assuming the ship moves along the *x*-axis, defining the fluid velocity potential as Φ*_ship_*, the ship speed as *V_s_*, and the gravitational acceleration as *g*, which is taken from the value of 9.81 m/s^2^. Then, the relationship between the Kelvin wake elevation surface *Z_ship_* of the ship and Φ*_ship_* is

(1) $$Z_{\mathrm{ship}}=\frac{V_{s}}{g}\frac{\partial \Phi_{\mathrm{ship}}}{\partial x}$$

Redefine *B* as the width of the ship, *L* as the length of the ship, *D* as the depth of the ship draft, and Re[·] as the real part. The Φ_ship_ is approximately [11]

(2) Φship(x,y,z)=−16BLVsFr6πRe[∫0∞C(τ,x,z)eiyτdτ]

Define *θ* as the angle of wave propagation, and replace the variable integration with *τ*. *F*r is the dimensionless Froude number, which is used to determine the resistance of a ship. When *F*r = 0.5, it will have a classical Kelvin angle [26]. The other parameters are

(3) $$C(\tau ,x,z)=(1-e^{-v\alpha (\tau )D})e^{zv\alpha (\tau )}\cos(xv\alpha^{1/2}(\tau ))\frac{\sin[\beta (\tau )]-\beta (\tau )\cos[\beta (\tau )]}{\alpha^{3/2}(\tau )\sqrt{1/4+\tau^{2}/v^{2}}}$$

(4) $$\tau =v\sqrt{\sec^{2}\theta -\sec\theta },\theta \in (-\pi /2,\pi /2)$$

(5) $$v=\frac{g}{V_{s}^{2}}$$

(6) $$F_{r}=\frac{V_{s}}{\sqrt{gL}}$$

(7) $$\alpha (\tau )=\frac{1+\sqrt{1+4\tau^{2}/v^{2}}}{2}$$

(8) $$\beta (\tau )=\frac{\sqrt{\alpha (\tau )}}{2F_{r}^{2}}$$

**Figure 1:**
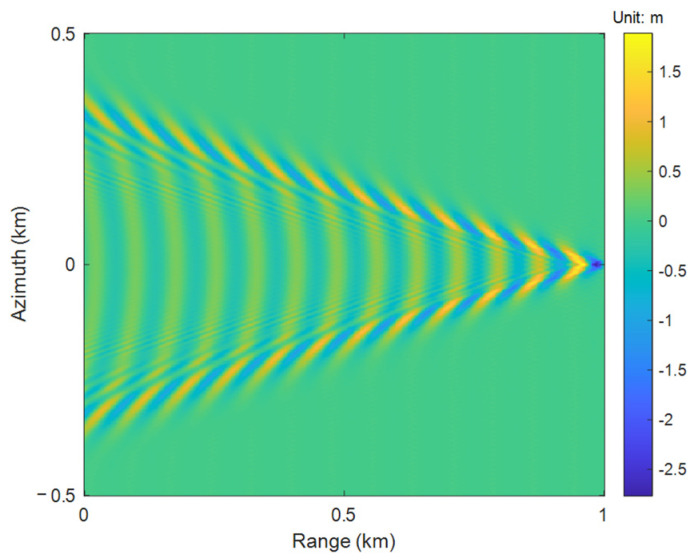
Simulation of Kelvin wake wave height.

The simulation result of Kelvin wake wave height in a 1 km field of view is shown in Figure 1. The parameters [25] of the ship model are shown in Table 1.

### 2.2. Model of Sea-Ship Surface Height

The sea states directly affect the inversion of wave height of the Kelvin wake in the marine environments. Therefore, by establishing a sea surface model, the wave height of the Kelvin wake under different sea states can be simulated. The assumption of water is the same as that of the Kelvin wake model, and the motion of water is irrotational. The three-dimensional (3D) motion of waves is established through a random phase model of the sum of many independent harmonics. Assuming that they propagate along the direction of *θ_j_* in *x*-*y* and *t* spaces, with an amplitude of *A_ij_* following a Rayleigh distribution, a random phase *r_ij_* ∈ (0, 2π) following a uniform distribution, *ω_i_* being the angular wave frequencies, and the sea wave height of *Z*_sea_ being expressed as follows:

(9) $$Z_{\mathrm{sea}}(x,y,z,t)=\sum_{i}\sum_{j}A_{ij}\cos[k_{i}(x\cos\theta_{j}+y\sin\theta_{j})-\omega_{i}t+r_{ij}]$$

Define *k*_i_ as angular wave numbers, and express *ω_i_* as

(10) $$\omega_{i}=\sqrt{gk_{i}}$$

Define *S*(*k_i_*) as the spectrum density, *D*(*k_i_*,*θ_j_*) as the angular wave spreading function, Δ*k_i_* and Δ*θ_j_* corresponding to the sampling intervals of *k_i_* and *θ_j_*, and *A_ij_* as follows:

(11) $$A_{ij}=\sqrt{2S(k_{i})D(k_{i},\theta_{j})\Delta k_{i}\Delta \theta_{j}}$$

Using the Joint North Sea Wave Project (JONSWAP) spectrum to describe sea states and gravity waves, the backscattering of the waves and propagating wakes is relatively large, resulting in better visualization and detectability of the wakes [25]. The *S*(*k_i_*) is as follows:

(12) $$S(k_{i})=\frac{\alpha }{2}{k_{i}}^{-3}e^{-1.25{\left(k_{i}/k_{p}\right)}^{-2}}e^{\ln(\gamma )\exp[-{\left(\sqrt{k_{i}/k_{p}}-1\right)}^{2}/(2\sigma^{2})]}$$

Among them, *F* is defined as the fetch length, *U*_10_ is the wind velocity at 10 m above the mean sea surface, and *α* [27] and *γ* [28] are expressed as follows:

(13) $$\alpha =0.0817{\left(gF/{U_{10}}^{2}\right)}^{-2/7}$$

(14) $$\gamma =7{\left(gF/{U_{10}}^{2}\right)}^{-1/7}$$

*k_p_* is the peak wave number, expressed as follows [25]:

(15) $$k_{p}={\left[7\pi \frac{\sqrt{g}}{U_{10}}{\left(\frac{{U_{10}}^{2}}{gF}\right)}^{0.33}\right]}^{2}$$

Parameter σ is related to the values of *k_i_* and *k_p_*, with the following values:

(16) $$\sigma =\left\{\begin{array}{c} 0.07,k_{i}\leq k_{p}\\ 0.09,k_{i}\geq k_{p} \end{array}\right.$$

*S*(*k_i_*) is an ID omnidirectional spectrum. In order to construct a 2D spectrum of the superimposed state of the wave energy transfer components along the wind direction, (11) also has an important parameter of *D*(*k_i_*,*θ_j_*). Higher-order functions usually have better directivity for wave propagation, and the most widely used one is the cosine-type spreading function based on empirical field measurements of pitch-and-roll buoys by Longuet-Higgins et al. [29]:

(17) $$D(\theta_{j})=\frac{\Gamma (S+1)}{2\sqrt{\pi }\Gamma (S+0.5)}\cos^{2S}(\frac{\theta_{j}-\theta_{w}}{2})$$

where Γ(·) is the gamma function, the *S* controls that the width of the function is related to *k_i_*, and *θ_w_* is the direction of the mean sea breeze. The simulation parameters [25] are shown in Table 2.

**Figure 2:**
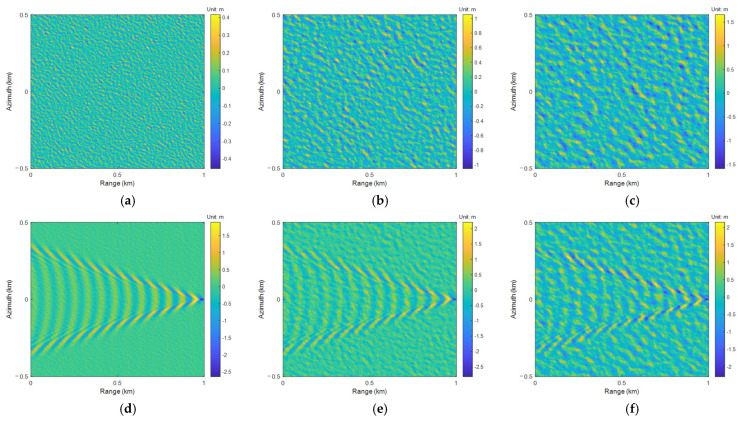
Sea wave height: (**a**) *U*_10_ = 3 m/s; (**b**) *U*_10_ = 8 m/s; (**c**) *U*_10_ = 13 m/s. Sea-ship surface height: (**d**) *U*_10_ = 3 m/s; (**e**) *U*_10_ = 8 m/s; (**f**) *U*_10_ = 13 m/s.

In the marine environment, the wake wave height of the ship is bound to be partially covered up, so the presence of waves can be regarded as noise. When the wind speed is about 3 m/s or lower, the Kelvin wake system (divergent and transverse waves) can be best observed, as these levels create a very calm sea surface [30]. When the wind speed is greater than 13 m/s, the visibility of the Kelvin wake system is poor. According to (9)–(17), when other simulation parameters are determined, the $\exp[-{\left(\sqrt{k_{i}/k_{p}}-1\right)}^{2}/(2\sigma^{2})]\approx 0$ in (12) under this scale simulation condition, so *Z*_sea_∝*K*_1_*U*_10_^2/7^exp(−*K*_2_*U*_10_^−17/25^) (*K*_1_ and *K*_2_ are coefficients greater than 0). It indicates that as the wind velocity increases, the wave height also increases, and the two are positively correlated. For the wind velocity range of 3–13 m/s, the median value of 8 m/s is taken (refer to Section 4.3 for relevant analysis and discussion). Using the different wind velocity parameters *U*_10_ of 3 m/s, 8 m/s, and 13 m/s to simulate different noise levels, respectively, as shown in Figure 2a–c.

Superimpose the sea wave height with the Kelvin wake wave height of the ship, and the results of the sea-ship surface height are shown in Figure 2d–f.

### 2.3. Model of Simulating Phase

**Figure 3:**
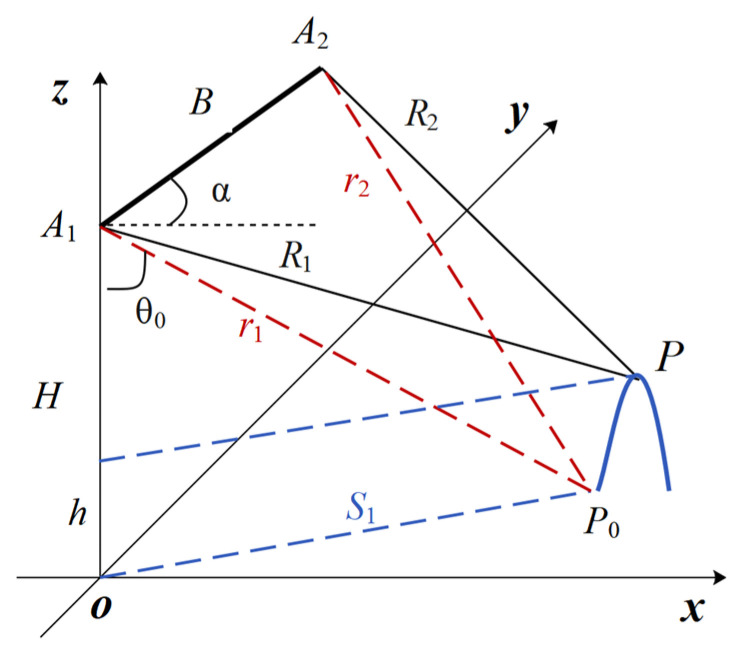
The geometric model of topographic phase for InSAR.

The variation in wave height in radar images is manifested as the distance difference in the radar received signal, which is a function of the phase difference between the receiving antennas in InSAR [31], and Δφ is known as the interferometric phase. Simulate the phase by establishing the geometric relationship between the radar observation position and the image, as shown in Figure 3.

The *x*-axis and *y*-axis represent the range and azimuth directions, respectively, while the *z*-axis represents the height direction. Radar represents the radar position as (0, 0, *H*), *P* represents the target’s position, the distance from antenna *A*_1_ is *R*_1_, and the distance from antenna *A*_2_ is *R*_2_. The equidistant projection of *P* onto the reference ellipsoid is *P*_0_, so *r*_1_ = *R*_1_. The wave height *h*, which is the sea-ship surface height mentioned in the previous section, causes an increase in *δθ* in the radar field of view, so *r*_2_ ≠ *R*_2_. Therefore, the slant range difference between the two radar images is

(18) $$\delta R=R_{1}-R_{2}$$

Define *B* as the spatial baseline of two antennas, where *α* is the angle between *B* and the horizontal direction (baseline-orientation angle), approximately derived from the slant range difference relationship:

(19) $$\delta R=B\sin(\theta_{0}+\delta \theta -\alpha )$$

Due to the fact that the *R* and *H* are much greater than the *h* of the observation target, the above equation is approximately expanded as follows:

(20) $$\delta R=B\sin(\theta_{0}-\alpha )+B\cos(\theta_{0}-\alpha )\delta \theta$$

The first term represents the slant range difference caused by the reference ellipsoid, and the second term represents the slant range difference caused by the target elevation.

*λ* is the working wavelength of the radar. Therefore, the second term is converted into an interferometric phase model to obtain the Δφ expression:

(21) $$\Delta \varphi =-4\pi B\cos(\theta_{0}-\alpha )\delta \theta /\lambda$$

From the trigonometric relationship, it can be concluded that

(22) $$\cos(\theta_{0})=H/R_{1}$$

(23) $$\cos(\theta_{0}+\delta \theta )=(H-h)/R_{1}$$

Considering the extremely small *δθ*, it can be concluded that

(24) $$\delta \theta =h/(R_{1}\sin\theta )$$

By substituting (24) into (21), the following can be obtained:

(25) $$\Delta \varphi =-4\pi B\cos(\theta_{0}-\alpha )\cdot h/\left(\lambda R_{1}\sin\theta_{0}\right)$$

Design *B*, according to the optimal baseline *B_opt_* [32], with the formula as follows:

(26) $$B_{opt}=[1-\gamma_{opt}(1+{\mathrm{SNR}}^{-1})]\lambda R_{1}\tan\theta_{0}/\rho_{r}$$

**Figure 4:**
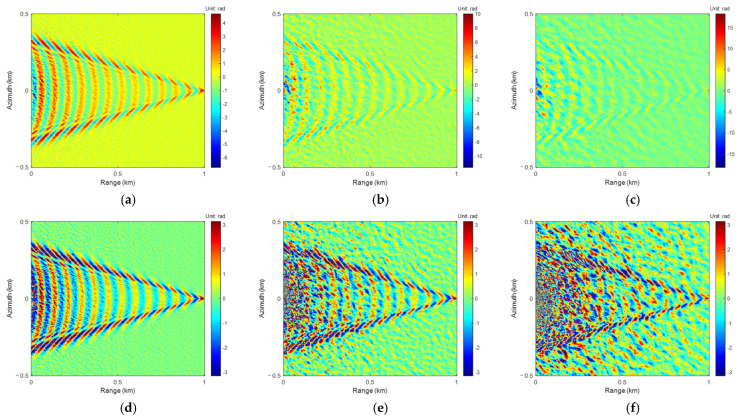
Simulation of true phase: (**a**) *U*_10_ = 3 m/s; (**b**) *U*_10_ = 8 m/s; (**c**) *U*_10_ = 13 m/s. Simulation of interferometric phase: (**d**) *U*_10_ = 3 m/s; (**e**) *U*_10_ = 8 m/s; (**f**) *U*_10_ = 13 m/s.

Among them, *γ_opt_* = 0.618–1.171 SNR^−1^ is the optimal correlation coefficient, SNR is the Signal-to-Noise Ratio, and *ρ_r_* is the intrinsic range resolution. *f* is the radar frequency, *c* is the speed of light, and *λ* = *c*/*f*. The simulation parameters of airborne SAR are shown in Table 3. From height to phase, the results are shown in Figure 4.

## 3. Methods and Results of PU Algorithms

### 3.1. 4-FFT Algorithm

#### 3.1.1. Principle of 4-FFT Algorithm

The interferometric phase obtained from two SAR images is wrapped in [−π,π), and an interval and differs from the true phase by an integer multiple *n* of 2π, expressed as follows:

(27) ϕ(i,j)=ψ(i,j)+2πn(i,j)

Among them, *ϕ*(*i*,*j*) represents the unwrapped phase, *ψ*(*i*,*j*) represents the wrapped phase, and (*i*,*j*) represents the real domain pixel coordinates. lm represents taking the imaginary part [33], and the *ϕ*(*i*,*j*) and *ψ*(*i*,*j*) relationship [34]:

(28) $$e^{i\psi }=e^{i(\phi -2\pi n)}=e^{i\phi }$$

(29) Im∇2eiψeiψ=Im∇2(cosψ+isinψ)cosψ+isinψ

(30) Im∇2eiϕeiϕ=Im[−∇ϕ2+i∇2ϕ]

(31) $$\nabla^{2}\phi =\cos\psi \cdot \nabla^{2}(\sin\psi )-\sin\psi \cdot \nabla^{2}(\cos\psi )$$

Generally, PU algorithms are used to solve for phase jumps that occur when the phase difference between adjacent pixels exceeds a certain threshold. The operation of finding the difference is equivalent to the process of finding the partial derivative. So, by using 2D Laplace to approximate the above process, it is expressed as follows:

(32) $$n(i,j)=\nabla^{-2}[\nabla^{2}\phi (i,j)-\nabla^{2}\psi (i,j)]/(2\pi )$$

Among them, ∇^2^ and ∇^−2^, respectively, represent the 2D Laplacian operator and its inverse operator, (*i*,*j*) and (*k*,*l*) represent the pixel coordinates in the Real and Fourier domains, and *M* and *N* represent the number of rows and columns in the input image. They are also, respectively, shown by Fast Fourier Transform (FFT) and Inverse FFT (IFFT):

(33) $$\nabla^{2}f(i,j)=-4\pi^{2}\mathrm{IFFT}[(k^{2}+l^{2})\mathrm{FFT}[f(i,j)]]/(MN)$$

(34) $$\nabla^{-2}g(i,j)=-MN\mathrm{IFFT}[\mathrm{FFT}[g(i,j)]/(k^{2}+l^{2})]/(4\pi^{2})$$

By combining (27) and (31)–(34), we obtain:

(35) $$\begin{array}{c} \phi =\psi +\mathrm{IFFT}[\mathrm{FFT}[\cos\psi \{\mathrm{IFFT}[(k^{2}+l^{2})\mathrm{FFT}[\sin\psi ]]\}-\sin\psi \{\mathrm{IFFT}[(k^{2}+l^{2})\mathrm{FFT}[\cos\psi ]]\}\\ -\mathrm{IFFT}[(k^{2}+l^{2})\mathrm{FFT}[\psi ]]]/(k^{2}+l^{2})] \end{array}$$

This process requires four FFTs and IFFTs, so it is called the 4-FFT algorithm. Centering the zero frequency during the process can maintain phase continuity and thus enhance the accuracy of interferometric measurements. It is not based on any assumptions, avoiding noise statistics related to wrapped phase data [35]. It is not only used to solve differential equations, but also to calculate derivatives instead of finite differences, avoiding nearest neighbor operations on adjacent pixel noise [36]. When the input is a periodic function, boundary condition issues can be ignored. So, it is necessary to perform periodic extension on the wrapped phase of the input [37]. Research has shown that mirror padding and periodic extension can ensure the smoothness of the extended boundary, whether for axisymmetric and non-axisymmetric matrices [38].

#### 3.1.2. Results of 4-FFT Algorithm

**Figure 5:**
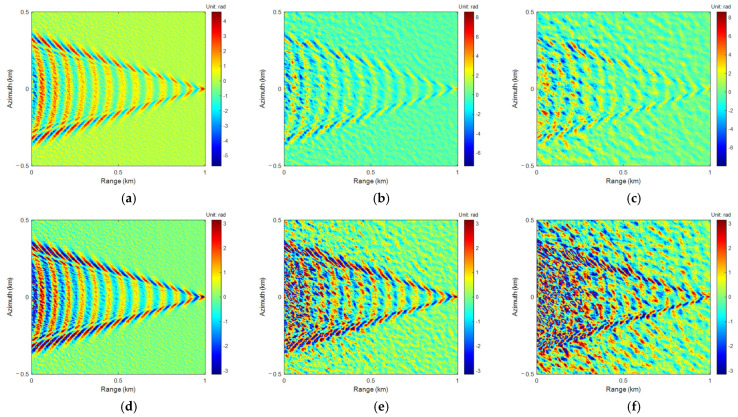
The results of 4-FFT PU: (**a**) *U*_10_ = 3 m/s; (**b**) *U*_10_ = 8 m/s; (**c**) *U*_10_ = 13 m/s. The results of 4-FFT phase rewrapping: (**d**) *U*_10_ = 3 m/s; (**e**) *U*_10_ = 8 m/s; (**f**) *U*_10_ = 13 m/s.

The phase models of sea-ship surface height simulated in different wind velocities were wrapped to generate interferometric phase images. The 4-FFT PU algorithm was applied for PU and phase rewrapping. The results are shown in Figure 5.

As wind velocities increase, sea surface background noise gradually increases, and the differences between the PU and phase rewrapping results and the true phase and interferometric phase become larger, respectively.

### 3.2. Proposed ALoG Algorithm

#### 3.2.1. Principle of Improved LoG Algorithm

Although the 4-FFT can achieve high unwrapping accuracy, applying mirror processing to the boundaries of the input image is only an approximation, and the presence of noise can also introduce unwrapping errors. Therefore, this paper proposes combining the Gaussian function and the Laplacian operator to develop the LoG PU algorithm, which can effectively detect edges and details in images while suppressing noise. Define σ as the standard deviation of the Gaussian function, and the Gaussian function *G*(*i*,*j*) is expressed as

(36) $$G(i,j)=\frac{1}{2\pi \sigma^{2}}e^{-\frac{i^{2}+j^{2}}{2\sigma^{2}}}$$

For Gaussian functions, their Fourier Transform has special properties:

(37) $$\mathrm{FFT}[G(i,j)]=e^{-2\pi^{2}\sigma^{2}\left(\frac{k^{2}}{N^{2}}+\frac{l^{2}}{M^{2}}\right)}$$

In the determined sliding window size, the variance within the window is calculated as the input parameter for the central pixel, allowing for the adjustment of the variance *σ*^2^ to smooth the image while preserving important edges and details information. We defined $\nabla_{LoG}^{2}$ and $\nabla_{LoG}^{-2}$ as the Gaussian Laplacian operator and the Gaussian Laplacian inverse operator, represented by FFT and IFFT:

(38) $$\nabla_{LoG}^{2}f(i,j)=-\frac{4\pi^{2}}{MN}\mathrm{IFFT}[(k^{2}+l^{2})e^{-2\pi^{2}\sigma^{2}\left(\frac{k^{2}}{N^{2}}+\frac{l^{2}}{M^{2}}\right)}\mathrm{FFT}[f(i,j)]]$$

(39) $$\nabla_{LoG}^{-2}g(i,j)=-\frac{MN}{4\pi^{2}}\mathrm{IFFT}\left[e^{-2\pi^{2}\sigma^{2}\left(\frac{k^{2}}{N^{2}}+\frac{l^{2}}{M^{2}}\right)}\frac{\mathrm{FFT}[g(i,j)]}{\left(k^{2}+l^{2}\right)}\right]$$

Replace (33) and (34) with (38) and (39), and join (27), (31) and (32) to obtain:

(40) $$\phi =\psi +\mathrm{IFFT}\left[\mathrm{FFT}\left[\begin{array}{c} \cos\psi \left\{\mathrm{IFFT}\left[(k^{2}+l^{2})e^{-2\pi^{2}\sigma^{2}\left(\frac{k^{2}}{N^{2}}+\frac{l^{2}}{M^{2}}\right)}\mathrm{FFT}\left[\sin\psi \right]\right]\right\}\\ -\sin\psi \left\{I\mathrm{FFT}\left[(k^{2}+l^{2})e^{-2\pi^{2}\sigma^{2}\left(\frac{k^{2}}{N^{2}}+\frac{l^{2}}{M^{2}}\right)}\mathrm{FFT}\left[\cos\psi \right]\right]\right\}\\ -\mathrm{IFFT}\left[(k^{2}+l^{2})e^{-2\pi^{2}\sigma^{2}\left(\frac{k^{2}}{N^{2}}+\frac{l^{2}}{M^{2}}\right)}\mathrm{FFT}\left[\psi \right]\right] \end{array}\right]/(k^{2}+l^{2})e^{-2\pi^{2}\sigma^{2}\left(\frac{k^{2}}{N^{2}}+\frac{l^{2}}{M^{2}}\right)}\right]$$

#### 3.2.2. Adaptive σ

In Gaussian functions, the selection of variance σ^2^ can affect image quality. Larger values lead to a wider Gaussian function used for smoothing, weakening the edge information. Choosing smaller values leads to a narrower Gaussian function used for highlighting edges, preserving detailed information. The calculation of variance *σ*^2^ varies depending on the size of the sliding window; therefore, an adaptive algorithm is introduced. Determine the optimal standard deviation σ by solving for the minimum sum of squared errors. The function expression is

(41) $$\sigma_{opt}(i,j)=\min[\varepsilon_{\sigma_{2H+1}}^{2}(i,j)]$$

Among them, $\varepsilon_{\sigma_{2H+1}}^{2}(i,j)$ represents the pixel in the *i* row and *j* column. Calculate the standard deviation *σ* when the sliding window size is 2*H* + 1 (*H* = 1, 2, 3…), and then calculate the sum of squared errors between the rewrapping unwrapped phase and the original wrapped phase. Finally, based on the LoG, the optimal PU result is obtained from the optimal standard deviation σ. This method is called the ALoG PU algorithm.

#### 3.2.3. Results of LoG and ALoG Algorithms

**Figure 6:**
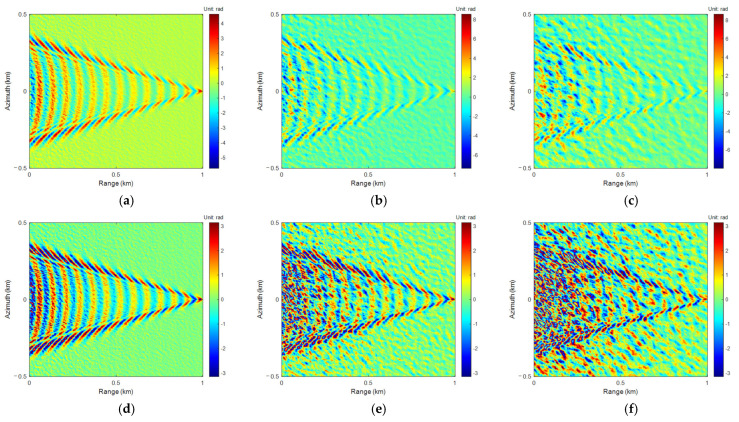
The results of LoG PU: (**a**) *U*_10_ = 3 m/s; (**b**) *U*_10_ = 8 m/s; (**c**) *U*_10_ = 13 m/s. The results of LoG phase rewrapping: (**d**) *U*_10_ = 3 m/s; (**e**) *U*_10_ = 8 m/s; (**f**) *U*_10_ = 13 m/s.

**Figure 7:**
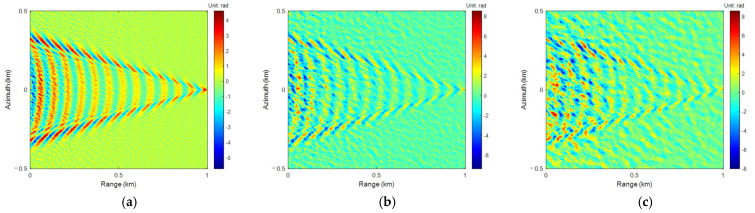

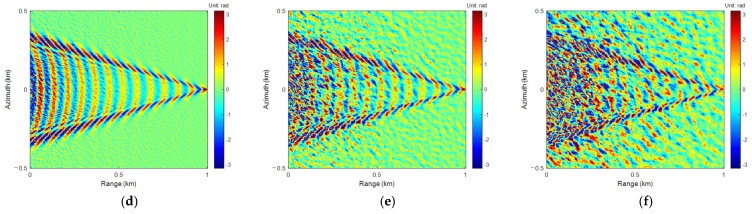
The results of ALoG PU: (**a**) *U*_10_ = 3 m/s; (**b**) *U*_10_ = 8 m/s; (**c**) *U*_10_ = 13 m/s. The results of ALoG phase rewrapping: (**d**) *U*_10_ = 3 m/s; (**e**) *U*_10_ = 8 m/s; (**f**) *U*_10_ = 13 m/s.

Similarly to the 4-FFT PU algorithm, the interferometric phases in Figure 4d–f were unwrapped using LoG and ALoG PU algorithms, and the relevant results are shown in Figure 6 and Figure 7.

Comparing Figure 5, Figure 6 and Figure 7, there is not much difference in the PU and phase rewrapping results of the 4-FFT, LoG, and ALoG algorithms, but the PU result of the ALoG algorithm has a larger unwrapping range at *U*_10_ of 13 m/s, indicating it is better.

Then, quantitatively evaluate the effectiveness of the PU algorithms. The simulated true phase is known, and the quality of PU results can be evaluated based on these four indicators: Correlation (Cor) between unwrapped phase and true phase, Root Mean Square Error (RMSE), SNR, and Peak SNR (PSNR). At the same time, these four indicators (represented as Re-Cor, Re-RMSE, Re-SNR, and Re-PSNR) can also be used to compare the rewrapped phase with the interferometric phase, as shown in Table 4.

When *U*_10_ is 3 m/s, the sea surface background noise is relatively weak. Apart from the Cor indicator, the LoG algorithm exhibits improvements in all other indicators compared to the 4-FFT algorithm. As the wind velocity increases, the sea surface background noise also increases. When *U*_10_ is 8 m/s, the RMSE of the LoG algorithm is comparable to that of the 4-FFT algorithm, but the LoG algorithm falls behind in other indicators. The wind velocity is further strengthened, and when *U*_10_ is 13 m/s, the four rewrapping indicators of the LoG algorithm are better than those of the 4-FFT algorithm, suggesting a certain degree of enhancement over the 4-FFT algorithm, albeit with instability. Conversely, as the wind velocity gradually increases and the sea surface background noise also gradually increases, all indicators of the ALoG algorithm demonstrate superiority, highlighting its improved effectiveness and having certain noise resistance and robustness.

Although the ALoG algorithm performs the best in various indicators, the improvement effect compared to the 4-FFT and LoG algorithms is not significant. In addition, compared with the true phase in Figure 4a–c, there is still a certain gap between the ALoG and the true phase in the unwrapping range, so further optimization is needed.

### 3.3. Principle and Method of Iteration

#### 3.3.1. Principle of Iteration

In order to minimize PU errors as much as possible, error iteration can be used to ensure high unwrapping accuracy while also being applicable to low SNR. The principle is to use the main value of the unwrapped error as input for PU, add the true phase of the unwrapped error to the last result of PU, and repeat the above process until a certain condition is met. By continuously compensating for the unwrapped phase, the phase errors become smaller and the unwrapped phase becomes closer to the true phase.

Assuming *ϕ*(*i*,*j*) is an unwrapped phase, Δ*φ*(*i*,*j*) is denoted as unwrapping error, expressed as follows:

(42) ϕ(i,j)=φ(i,j)+Δφ(i,j)

The true phase is unknown, the unwrapping error is also unknown, and the range change may exceed 2π. Therefore, the unwrapping error can be represented by the main value of the unwrapping error of Δ*φ*_m_(*i*,*j*):

(43) $$\Delta \varphi (i,j)=\Delta \varphi_{m}(i,j)+2\pi n_{m}(i,j)$$

Combining (42) and (43):

(44) $$\phi (i,j)=\varphi (i,j)+\Delta \varphi_{m}(i,j)+2\pi n_{m}(i,j)$$

The difference between (44) and (27) shows the following:

(45) $$\psi (i,j)-\varphi (i,j)=\Delta \varphi_{m}(i,j)+2\pi n_{m}(i,j)-2\pi n(i,j)$$

W[·] is taken as the main value of the phase:

(46) $$W[\psi (i,j)-\varphi (i,j)]=W[\Delta \varphi_{m}(i,j)+2\pi n_{m}(i,j)-2\pi n(i,j)]=\Delta \varphi_{m}(i,j)$$

**Figure 8:**
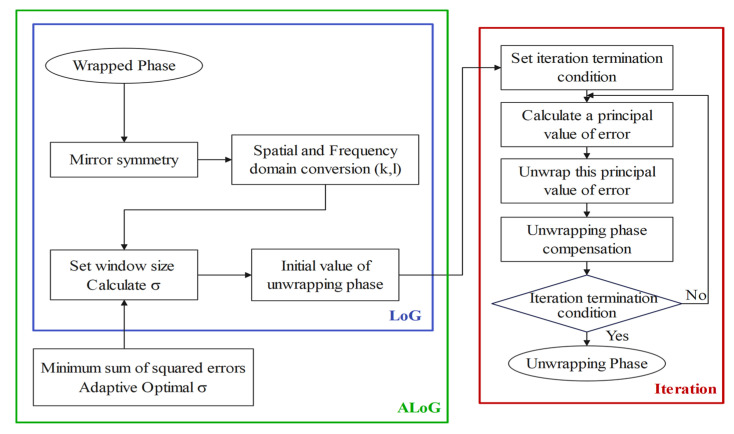
Flowchart of PU algorithms.

The unwrapped phase compensates closer to the true phase by unwrapping the main value of the unwrapped error. The iterative process can be expressed as follows:

(47) $$\left\{\begin{array}{l} \phi^{t+1}(i,j)=\phi^{t}(i,j)+\mathrm{Unwrapping}[\Delta \varphi_{m}^{t}(i,j)]\\ \Delta \varphi_{m}^{t}(i,j)=W[\psi (i,j)-\phi^{t}(i,j)] \end{array}\right.$$

where $Unwrapping\left[\cdot \right]$ means different PU algorithms, t means the number of iterations, which is set to 50 by calculating the percentage that tends to saturate, and the iteration termination condition is set to the proportion of the residual number in the image is less than 0.01%. The algorithm flowchart is shown in Figure 8.

#### 3.3.2. Results of Iteration

**Figure 9:**
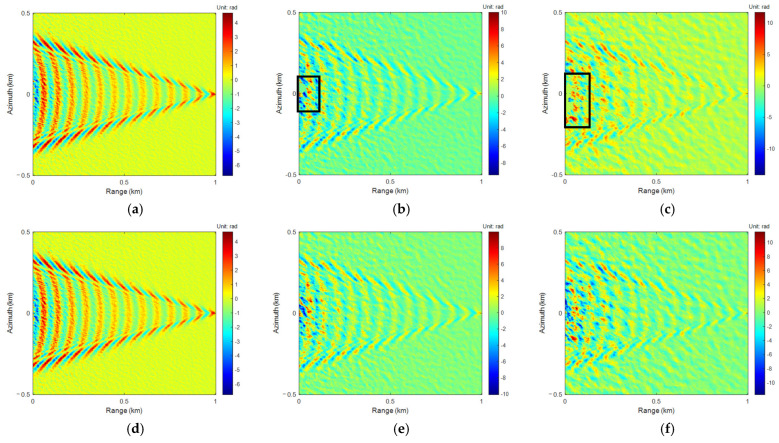
The results of Goldstein PU: (**a**) *U*_10_ = 3 m/s; (**b**) *U*_10_ = 8 m/s; (**c**) *U*_10_ = 13 m/s. The results of ALoGI PU: (**d**) *U*_10_ = 3 m/s; (**e**) *U*_10_ = 8 m/s; (**f**) *U*_10_ = 13 m/s.

**Figure 10:**
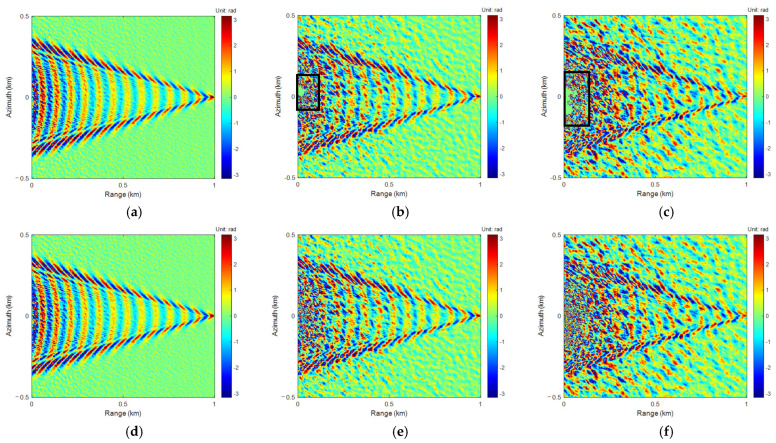
The results of Goldstein phase rewrapping: (**a**) *U*_10_ = 3 m/s; (**b**) *U*_10_ = 8 m/s; (**c**) *U*_10_ = 13 m/s. The results of ALoGI phase rewrapping: (**d**) *U*_10_ = 3 m/s; (**e**) *U*_10_ = 8 m/s; (**f**) *U*_10_ = 13 m/s.

The Goldstein [39] branch cuts method is a classic algorithm based on path tracing for local phase unwrapping. It involves selecting an appropriate path and utilizing the phase gradient between adjacent pixels for phase integration. The integration is related to the phase gradient, but not to the path it passes through. Starting from any point not on the branch tangent, the flood inundation method is employed to integrate the entangled phase gradient point by point. Based on the initial value of the unwrapping phase of the ALoG algorithm, the ALoG was used for iteration, and the results of the Goldstein and ALoG iteration (ALoGI) algorithms are shown in Figure 9 and Figure 10.

In the process of PU in Goldstein, it does not cross the branch tangent, so the integration of adjacent pixel points with an absolute gradient value of half a cycle can be limited to the branch, effectively suppressing the propagation of phase errors. However, for areas with dense residual points, it may form a closed network isolated from other areas, resulting in islands that cannot be unwrapped and rendering the algorithm completely ineffective, as shown in the black box areas in Figure 9b,c and Figure 10b,c. In addition, improper setting of branch tangents or undersampling of regions can cause errors to jump throughout the entire cycle, leading to the propagation of phase errors. Overall, the ALoGI algorithm performs PU on each pixel compared to the Goldstein algorithm, obtains a larger phase unwrapping range compared to the ALoG algorithm, and is closer to the true phase, resulting in better overall performance.

Similarly to Table 4, the indicators corresponding to the PU and phase rewrapping of Goldstein and ALoG algorithms were separately calculated to better evaluate the advantages and disadvantages of the PU algorithms. The results are shown in Table 5. Compared with the ALoG algorithm, when *U*_10_ is 3 m/s, the RMSE, SNR, and PSNR indicators of the Goldstein algorithm have been improved to a certain extent. The Re-RMSE is reduced by 77.12%, the Re-SNR is improved by 130.21%, and the Re-PSNR is improved by 21.56%, indicating that the Goldstein algorithm has a better unwrapping effect under the noise corresponding to this wind velocity. The ALoGI algorithm effectively improved all indicators and performed the best, especially when RMSE and Re-RMSE were significantly reduced by 97.60% and 84.77%, respectively. SNR and Re-SNR were significantly improved by 140.13% and 166.04%, respectively, while PSNR and Re-PSNR were improved by 44.64% and 27.49%, respectively, which were closer to the true phase.

When *U*_10_ is 8 m/s, the Goldstein algorithm encounters islanding areas that cannot be unwrapped, resulting in lower PU performance compared to the ALoG algorithm. However, the phase rewrapping performance is better than both the ALoG algorithm and the ALoGI algorithm. Therefore, when the true phase of radar measured is unknown, only the phase rewrapping performance can be compared. Although the Goldstein algorithm performs the best, there is actually a certain gap between it and the true phase. The four PU indicators of the ALoGI algorithm performed the best, and all four phase rewrapping indicators were effectively improved. Among them, RMSE and Re-RMSE decreased by 67.24% and 73.60%, respectively, and SNR and Re-SNR increased by 66.61% and 169.21%, respectively, with significant effects.

When *U*_10_ is 13 m/s, the Goldstein algorithm exhibits islanded regions that have not been correctly unwrapped, similar to *U*_10_ being 8 m/s. It also shows that the four PU indicators perform the worst while the four phase rewrapping indicators are the best, indicating that the reliability of the Goldstein algorithm is poor. All indicators of the ALoG algorithm have been effectively improved, especially in the reduction in RMSE and Re-RMSE and the improvement of SNR and Re-SNR, with some degree of improvement in Cor, Re-Cor, PSNR, and Re-PSNR, indicating the effectiveness of the proposed algorithm.

The ALoGI PU algorithm greatly improves the unwrapping accuracy through multiple iterations of phase compensation, and can still maintain high unwrapping accuracy in low SNR environments.

## 4. Discussion

### 4.1. PCA

**Figure 11:**
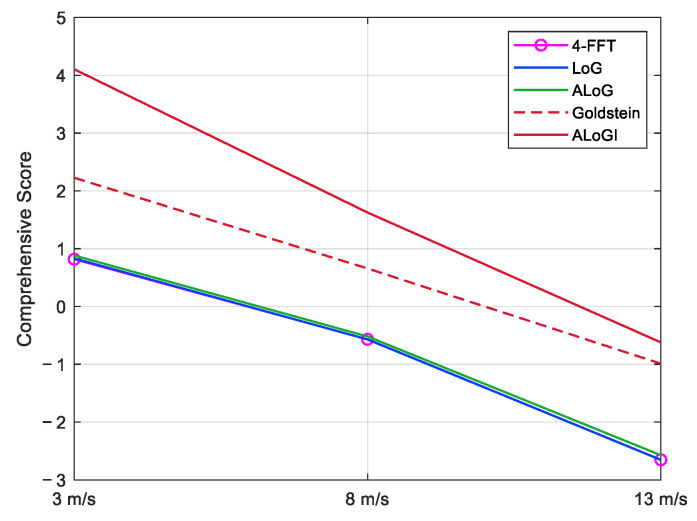
PCA scores in different wind velocity levels.

The PCA [40] reduces the complexity of high-dimensional data by transforming the original data into a new coordinate system, extracting important information, and visualizing data through principal component scores. In the evaluation of PU algorithms, the higher the Cor, the smaller the RMSE, and the higher the SNR and PSNR, the better the PU effect. Because there are a total of eight evaluation indicators, PCA was chosen for further visualization analysis of these five algorithms, with a contribution rate greater than 95%. The comprehensive score was calculated, and the results are shown in Table 6. Based on PCA scores, analyze the advantages and disadvantages of these PU algorithms under different wind velocities, as shown in Figure 11. According to the PCA score graph analysis, as the wind velocity increases, the sea surface background noise gradually increases. From a wind velocity of 3 m/s to a wind velocity of 13 m/s, the PCA comprehensive scores of all algorithms show a decreasing trend, with the percentage gradually decreasing in this order.

When the wind velocity is 3 m/s, the PCA scores of the 4-FFT, LoG, ALoG, Goldstein, and ALoGI algorithms increase in order. The first three algorithms have little difference in PCA scores, while the Goldstein and ALoGI algorithms have significantly better scores. The Goldstein algorithm has lower scores than the ALoGI algorithm, indicating the best performance of this algorithm. When the wind velocity is 8 m/s, the LoG algorithm scores the lowest, while the ALoG algorithm outperforms the first two, indicating that selecting adaptive variance has certain stability and noise resistance, and is effective for PU. But the first three algorithms still have little difference, with the Goldstein algorithm and the ALoGI algorithm showing significant improvement in scores under increased wind speed, with the ALoGI algorithm still having the highest score. When the wind velocity is 13 m/s, the performance of various algorithms is consistent with the environment with a wind speed of 8 m/s.

After iteration, the ALoGI algorithm scored significantly higher than the original algorithm. The ALoGI algorithm not only inherits the good stability of the ALoG algorithm itself, but also further enhances its noise resistance through iteration. The ALoGI algorithm has the highest PCA comprehensive score in each wind velocity condition, with the smallest percentage decrease, thus proving the superiority of this algorithm.

### 4.2. Profile Analysis

**Figure 12:**
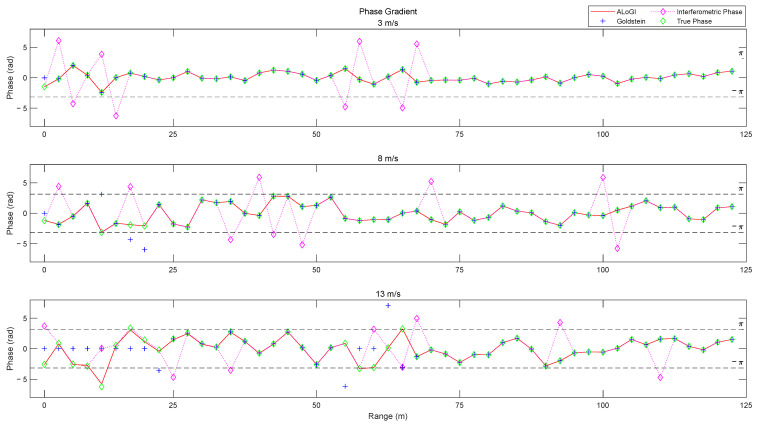
Profile analysis of phase gradient in finding different velocity levels.

PU is the recovery of phase jumps caused by the absolute difference in phase between adjacent pixels being greater than π, aiming to ensure the continuity of the true phase. The phase difference is the phase gradient. For the area where the Goldstein algorithm appears as an island in Figure 9, analyze the center of the interferometric phase gradient and PU gradient of the Goldstein and ALoGI algorithms along the range profile. The result is shown in Figure 12. For pixels with interferometric phase gradients greater than π or less than −π, PU is necessarily performed. Therefore, when the true phase gradient is between −π and π, PU can be considered correct, and the interferometric phase gradient is equal to the true phase gradient.

According to the analysis in Figure 12, it can be seen that the Goldstein algorithm effectively disentangles the positions of the interferometric phase gradients where phase jumps occur when *U*_10_ is 3 m/s, ensuring the continuity of the true phase. But the unwrapping phase gradient at the initial position is not equal to the interferometric phase gradient, which fails to reflect the true phase gradient. When *U*_10_ is 8 m/s, in addition to the phase problem at the initial position, there is an error in unwrapping at 17.5 m and a misunderstanding of the phase at 12.5 m and 20 m, all of which result in phase gradients exceeding the range of [−π, π). When *U*_10_ is 13 m/s, the phase gradient remains 0 rad from 0 m to 20 m due to the islanding phenomenon. There are phase gradient jumps at 22.5 m, 55 m, and 62.5 m. Due to error propagation, the true phase gradient cannot be reflected at 57.5 m to 60 m. Although it is consistent with the interferometric phase gradient at 65 m, it is different from the true phase gradient.

It is not difficult to know from the figure that when *U*_10_ is 3 m/s and 8 m/s, the true phase gradient is between −π and π, indicating that the true phase is continuous in both cases, satisfying the assumption of phase continuity. When *U*_10_ is 13 m/s, the true phase gradient at 12.5 m and 65 m exceeds the range of [−π, π), and the true phase is discontinuous. Therefore, in this case, the assumption of phase continuity is not satisfied. The ALoGI algorithm not only performs PU on pixels with phase jumps in the interferometric phase gradients of *U*_10_ at 3 m/s and 8 m/s, but also corresponds to the true phase gradient, indicating the effectiveness of the algorithm. In addition, when *U*_10_ is 13 m/s, the phase gradients at 12.5 m and 65 m correspond to the true phase gradients, indicating that the algorithm is not based on the assumption of phase continuity and further demonstrating its superiority.

### 4.3. Sea Surface Background

**Figure 13:**
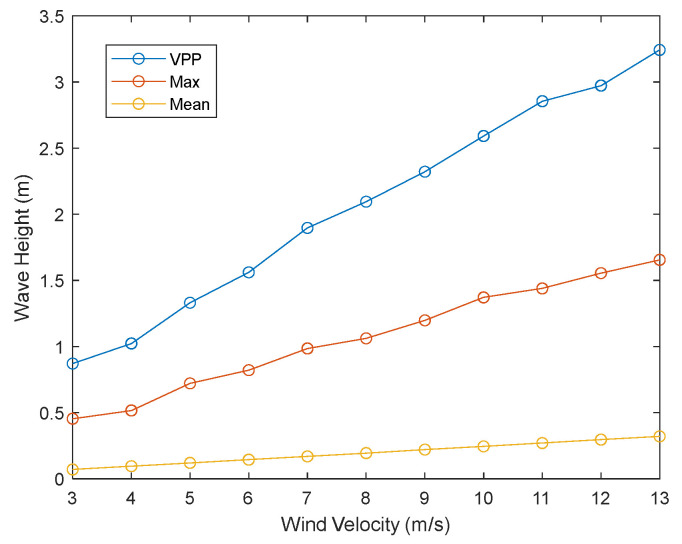
The influence of different wind velocity levels on wave height.

When researching the wave height of the target wake, the sea surface background obscures a portion of the target, and its presence is considered noise. Section 2.2. clearly states the parameters related to the sea surface background. When the sailing direction is consistent with the wind velocity direction, the transverse waves in the wake are more pronounced, which is also the basis for the parameter settings in this study. Through relevant simulations and research, it has been shown that sea surface wind velocity is the main factor affecting the visibility of Kelvin wake features in complex sea surface backgrounds. The aforementioned studies have shown that sea surface wind velocities between 3 and 13 m/s are the optimal range for studying Kelvin wakes. The wind velocity interval is 1 m/s, and the Peak-to-Peak Value (VPP), Maximum (Max), and mean wave height of each wind velocity within this range are calculated, as shown in Figure 13.

It is not difficult to see that as the wind velocity increases, the VPP, Max, and mean of the waves also increase, indicating that the fluctuations of the sea surface background waves become larger. In addition, these three curves are approximately linear, so the intermediate value of 8 m/s is selected for relevant research within the wind velocity range of 3–13 m/s.

In this paper, three different wind velocities of 3 m/s, 8 m/s, and 13 m/s were used to superimpose the simulated wake wave height to simulate the performance of the proposed ALoGI and PU algorithms under different sea surface background noise levels. However, the wake of ships in the sea is not a simple additive relationship, and there is a complex coupling relationship between waves, which requires more complex numerical models for simulation. This article did not delve into it in depth. In addition, the Kelvin wake behavior varies with different ship parameters, and when *F*_r_ > 0.5, the wake angle between sharp waves may decrease. And different combinations of ship length and velocity can also produce Kelvin wakes with the same waveform but different amplitudes.

Although there are numerous sea surface background models with different parameter settings, which may differ from the actual wake performance of ships in the sea, this paper focuses on the observation model of airborne SAR, simulates the interferometric phase of Kelvin wake wave height under different wind velocities, and applies the proposed ALoGI algorithm for PU. In the case where the sea surface background noise gradually covers the target wake, the algorithm improvement effect is significant and performs the best, indicating that the proposed algorithm has certain noise resistance and stability, and achieves the inversion of the ship wake wave height.

## 5. Conclusions

The advantages of airborne SAR, such as being real-time and portable, provide a measurement method for Kelvin wake wave height inversion from ships on the sea surface. The particularity of the complex form of radar images makes it possible to measure the wave height by phase interferometry. However, the entanglement of interferometric phase forces PU to become the focus and challenge of technology. The complexity of the sea waves partially submerges the Kelvin wake of ships, causing some noise pollution. Most algorithms based on the least squares still struggle to maintain both stability and noise resistance under the tested noise levels of the simulation. The PU experiments on the simulated sea-ship surface height model data show the following:

1. The proposed method improves the PU effect by introducing the Gaussian function into the Fourier expression of the 2D Laplacian.
2. The adaptive variance of the Gaussian function has good performance and high stability under the tested noise levels of the simulation.
3. The iterative algorithm plays a key role in the context of the tested noise levels of the simulation, significantly improving the performance of the algorithm and having good robustness. This highlights the superiority of the ALoGI algorithm.

## Figures and Tables

**Table 1:** Ship parameters.

Parameters	Values	Parameters	Values
*B*	50 m	*D*	3.5 m
*L*	6.5 m	*V*s	11 m/s (*F*r = 0.5)

**Table 2:** Simulation parameters of wave spectrum model.

Parameters	Values	Parameters	Values
*S*	7	*θ_w_*	45°
*F*	80 km	*t*	0

**Table 3:** The simulation parameters of airborne SAR.

Parameters	Values	Parameters	Values
*H*	3000 m	*B*	2.5 m
*f*	15 GHz	*c*	3 × 10^8^ m/s

**Table 4:** Statistics of PU results under different velocities.

		Cor	RMSE (rad)	SNR (dB)	PSNR (dB)	Re-Cor	Re-RMSE (rad)	Re-SNR (dB)	Re-PSNR (dB)
*U*_10_ = 3 m/s	4-FFT	0.9976	0.0664	22.4673	71.6810	0.9447	0.2794	9.6002	59.2049
	LoG	0.9975	0.0650	22.6559	71.8696	0.9450	0.2784	9.6339	59.2386
	ALoG	0.9977	0.0624	23.0089	72.2227	0.9476	0.2718	9.8407	59.4454
*U*_10_ = 8 m/s	4-FFT	0.9836	0.2211	14.4876	61.2401	0.8895	0.4707	6.6063	54.6760
	LoG	0.9835	0.2211	14.4853	61.2378	0.8893	0.4713	6.5958	54.6655
	ALoG	0.9838	0.2195	14.5496	61.3021	0.8953	0.4584	6.8359	54.9057
*U*_10_ = 13 m/s	4-FFT	0.9288	0.5900	8.5571	52.7132	0.8007	0.7582	4.0002	50.5356
	LoG	0.9286	0.5914	8.5376	52.6937	0.8013	0.7572	4.0117	50.5471
	ALoG	0.9296	0.5872	8.5990	52.7550	0.8118	0.7364	4.2533	50.7887

**Table 5:** Statistics of PU and phase rewrapping results under different wind velocities.

		Cor	RMSE (rad)	SNR (dB)	PSNR (dB)	Re-Cor	Re-RMSE (rad)	Re-SNR (dB)	Re-PSNR (dB)
*U*_10_ = 3 m/s	ALoG	0.9977	0.0624	23.0089	72.2227	0.9476	0.2718	9.8407	59.4454
	Goldstein	0.9988	0.0427	26.3067	75.5204	0.9973	0.0622	22.6547	72.2594
	ALoGI	0.9999	0.0015	55.2506	104.4643	0.9988	0.0414	26.1800	75.7846
*U*_10_ = 8 m/s	ALoG	0.9838	0.2195	14.5496	61.3021	0.8953	0.4584	6.8359	54.9057
	Goldstein	0.9756	0.2573	13.1689	59.9214	0.9943	0.1076	19.4268	67.4966
	ALoGI	0.9981	0.0719	24.2408	70.9933	0.9928	0.1210	18.4026	66.4723
*U*_10_ = 13 m/s	ALoG	0.9296	0.5872	8.5990	52.7550	0.8118	0.7364	4.2533	50.7887
	Goldstein	0.9181	0.6274	8.0232	52.1793	0.9894	0.1745	16.7601	63.2955
	ALoGI	0.9725	0.3674	12.6721	56.8281	0.9495	0.3819	9.9565	56.4919

**Table 6:** PCA scores of PU algorithms.

	4-FFT	LoG	ALoG	Goldstein	ALoGI
*U*_10_ = 3 m/s	0.8176	0.8326	0.8829	2.2274	4.1048
*U*_10_ = 8 m/s	−0.5677	−0.5705	−0.5192	0.6578	1.6260
*U*_10_ = 13 m/s	−2.6528	−2.6543	−2.5739	−0.9892	−0.6215

## Data Availability

The data presented in this study are available in this article.

## References

[1] Tings B., Pleskachevsky A., Wiehle S. (2023). Comparison of detectability of ship wake components between C-Band and X-Band synthetic aperture radar sensors operating under different slant ranges. ISPRS J. Photogramm. Remote Sens..

[2] Graziano M.D., Grasso M., D’Errico M. (2017). Performance analysis of ship wake detection on Sentinel-1 SAR images. Remote Sens..

[3] Hwang P.A., Sletten M.A., Toporkov J.V. (2010). A note on Doppler processing of coherent radar backscatter from the water surface: With application to ocean surface wave measurements. J. Geophys. Res. Ocean..

[4] Carrasco R., Streßer M., Horstmann J. (2017). A simple method for retrieving significant wave height from Dopplerized X-band radar. Ocean Sci..

[5] Carrasco R., Horstmann J., Seemann J. (2017). Significant wave height measured by coherent X-band radar. IEEE Trans. Geosci. Remote Sens..

[6] Liu P., Jin Y.Q. (2017). Simulation of synthetic aperture radar imaging of dynamic wakes of submerged body. IET Radar Sonar Navig..

[7] Tunaley J.K., Buller E.H., Wu K., Rey M.T. (1991). The simulation of the SAR image of a ship wake. IEEE Trans. Geosci. Remote Sens..

[8] Wang J.-K., Zhang M., Chen J.-L., Cai Z. (2016). Application of facet scattering model in SAR imaging of sea surface waves with Kelvin wake. Prog. Electromagn. Res. B.

[9] Thomson W. (1887). On ship waves. Proceedings of the Institution of Mechanical Engineers.

[10] Shemer L., Kagan L., Zilman G. (1996). Simulation of ship wakes image by an along-track interferometric SAR. Int. J. Remote Sens..

[11] Zilman G., Zapolski A., Marom M. (2014). On detectability of a ship’s Kelvin wake in simulated SAR images of rough sea surface. IEEE Trans. Geosci. Remote Sens..

[12] Jia Y., Liu S., Liu Y., Zhai L., Gong Y., Zhang X. (2024). Echo-Level SAR Imaging Simulation of Wakes Excited by a Submerged Body. Sensors.

[13] Hennings I., Romeiser R., Alpers W., Viola A. (1999). Radar imaging of Kelvin arms of ship wakes. Int. J. Remote Sens..

[14] Zhao Y., Zhang M., Zhao Y.-W., Geng X.-P. (2015). A bistatic SAR image intensity model for the composite ship–ocean scene. IEEE Trans. Geosci. Remote Sens..

[15] Bao M., Bruning C., Alpers W. (1997). Simulation of ocean waves imaging by an along-track interferometric synthetic aperture radar. IEEE Trans. Geosci. Remote Sens..

[16] Wilson M.B. (1971). A Michell Oseen-Flow Theory for Thin Ships. Ph.D. Thesis.

[17] Hasselmann K., Barnett T.P., Bouws E., Carlson H., Cartwright D.E., Enke K., Ewing J., Gienapp A., Hasselmann D., Kruseman P. (1973). Measurements of wind-wave growth and swell decay during the Joint North Sea Wave Project (JONSWAP). Ergaenzungsheft Zur Dtsch. Hydrogr. Z. Reihe A.

[18] Gao Z., Jia Y., Liu S., Zhang X. (2022). A 2-D frequency-domain imaging algorithm for ground-based SFCW-ArcSAR. IEEE Trans. Geosci. Remote Sens..

[19] Eshbaugh J.V., Frasier S.J. (2002). Measurement of sea surface displacement with interferometric radar. J. Atmos. Ocean. Technol..

[20] Zeng Y., Song C., Xu Z. (2022). Wave height estimation based on the phase time series of millimeter-wave radar. IEEE Geosci. Remote Sens. Lett..

[21] Sun J., Wang Y., Zhang J., Liang Y., Zhang G., Wan A., Zhang S., Ye Z., Zhou Y., Jing Q. (2024). 2-D Phase Unwrapping in DAS Based on Transport-of-Intensity-Equation: Principle, Algorithm and Field Test. J. Light. Technol..

[22] Zhao Z., Zhang H., Ma C., Fan C., Zhao H. (2020). Comparative study of phase unwrapping algorithms based on solving the Poisson equation. Meas. Sci. Technol..

[23] Wang K., Di J., Li Y., Ren Z., Kemao Q., Zhao J. (2020). Transport of intensity equation from a single intensity image via deep learning. Opt. Lasers Eng..

[24] Zuo C., Huang L., Zhang M., Chen Q., Asundi A. (2016). Temporal phase unwrapping algorithms for fringe projection profilometry: A comparative review. Opt. Lasers Eng..

[25] Rizaev I.G., Karakuş O., Hogan S.J., Achim A. (2022). Modeling and SAR imaging of the sea surface: A review of the state-of-the-art with simulations. ISPRS J. Photogramm. Remote Sens..

[26] Rabaud M., Moisy F. (2013). Ship wakes: Kelvin or Mach angle?. Phys. Rev. Lett..

[27] Rizaev I., Karakuş O., Hogan S.J., Achim A. The effect of sea state on ship wake detectability in simulated SAR imagery. Proceedings of the 2020 IEEE International Conference on Image Processing (ICIP).

[28] Mitsuyasu H., Tasai F., Suhara T., Mizuno S., Ohkusu M., Honda T., Rikiishi K. (1980). Observation of the power spectrum of ocean waves using a cloverleaf buoy. J. Phys. Oceanogr..

[29] Holthuijsen L.H. (2010). Waves in Oceanic and Coastal Waters.

[30] Panico A., Graziano M.D., Renga A. (2017). SAR-based vessel velocity estimation from partially imaged Kelvin pattern. IEEE Geosci. Remote Sens. Lett..

[31] Felguera-Martín D., González-Partida J.-T., Almorox-González P., Burgos-García M., Dorta-Naranjo B.-P. (2011). Interferometric inverse synthetic aperture radar experiment using an interferometric linear frequency modulated continuous wave millimetre-wave radar. IET Radar Sonar Navig..

[32] Rodriguez E., Martin J. (1992). Theory and design of interferometric synthetic aperture radars. Proceedings of the IEE Proceedings F (Radar and Signal Processing).

[33] Schofield M.A., Zhu Y. (2003). Fast phase unwrapping algorithm for interferometric applications. Opt. Lett..

[34] Hÿtch M., Snoeck E., Kilaas R. (1998). Quantitative measurement of displacement and strain fields from HREM micrographs. Ultramicroscopy.

[35] Zhai L., Zhang X. (2024). InSAR Phase Unwrapping Algorithm Based on 4-FFT and Multi Grid Optimization. J. Microw..

[36] Duan Y. (2017). Research on High-Accuracy InSAR Phase Unwrapping Technique via Iteration. Master’s Thesis.

[37] Zhang Z. (2012). Phase Unwrapping Algorithm in Digital Holographic Microscopy. Master’s Thesis.

[38] Fan X., Zhang X., Duan Y., Wei S. An Multi-baseline High-Accuracy 4-FFT Phase Unwrapping Method via Iterative. Proceedings of the 4th China High Resolution Earth Observation Conference.

[39] Deng X. (2013). Research on the Algorithm of Phase Unwrapping for Interferometric Synthetic Aperture Radar. Master’s Thesis.

[40] Kurita T. (2021). Principal component analysis (PCA). Computer Vision: A Reference Guide.

